# Genome-Wide Linkage Analysis Identifies Loci for Physical Appearance Traits in Chickens

**DOI:** 10.1534/g3.115.020883

**Published:** 2015-08-06

**Authors:** Yanfa Sun, Ranran Liu, Guiping Zhao, Maiqing Zheng, Yan Sun, Xiaoqiong Yu, Peng Li, Jie Wen

**Affiliations:** * Institute of Animal Science, Chinese Academy of Agricultural Sciences, Beijing 100193, People’s Republic of China; † College of Life Science, Longyan University, Longyan, Fujian 364012, People’s Republic of China; ‡ Fujian Provincial Key Laboratory of Preventive Veterinary Medicine and Biotechnology, Longyan University, Longyan, Fujian 364012, People’s Republic of China; § State Key Laboratory of Animal Nutrition, Beijing 100193, People’s Republic of China

**Keywords:** chicken, physical appearance traits, linkage analysis, feather-crested head, beard, comb, wattles, feathered feet

## Abstract

Physical appearance traits, such as feather-crested head, comb size and type, beard, wattles size, and feathered feet, are used to distinguish between breeds of chicken and also may be associated with economic traits. In this study, a genome-wide linkage analysis was used to identify candidate regions and genes for physical appearance traits and to potentially provide further knowledge of the molecular mechanisms that underlie these traits. The linkage analysis was conducted with an F2 population derived from Beijing-You chickens and a commercial broiler line. Single-nucleotide polymorphisms were analyzed using the Illumina 60K Chicken SNP Beadchip. The data were used to map quantitative trait loci and genes for six physical appearance traits. A 10-cM/0.51-Mb region (0.0−10.0 cM/0.00−0.51 Mb) with 1% genome-wide significant level on LGE22C19W28_E50C23 linkage group (LGE22) for crest trait was identified, which is likely very closely linked to the *HOXC8*. A QTL with 5% chromosome-wide significant level for comb weight, which partly overlaps with a region identified in a previous study, was identified at 74 cM/25.55 Mb on chicken (*Gallus gallus*; GG) chromosome 3 (*i.e.*, GGA3). For beard and wattles traits, an identical region 11 cM/2.23 Mb (0.0−11.0 cM/0.00−2.23 Mb) including *WNT3* and *GH* genes on GGA27 was identified. Two QTL with 1% genome-wide significant level for feathered feet trait, one 9-cM/2.80-Mb (48.0-57.0/13.40-16.20 Mb) region on GGA13, and another 12-cM/1.45-Mb (41.0−53.0 cM/11.37−12.82 Mb) region on GGA15 were identified. These candidate regions and genes provide important genetic information for the physical appearance traits in chicken.

There are 1077 reported local chicken (*Gallus gallus*; GG) breeds in the world (Rischkowsky and Pilling 2007). Physical appearance traits often are used to distinguish between breeds of chicken and include feather-crested head, comb size and type, beard, wattles size, and feathered feet. The physical appearance traits also are associated with growth, reproduction, and other important economic traits. For example, the crested head gene has significant negative effects on body weight (Magothe *et al.* 2010) and, comb size has an influence on sexual characters and viability (Von Schantz *et al.* 1995), whereas the rose-comb mutation causes both altered comb morphology and defective sperm motility (Imsland *et al.* 2012).

The genetic mechanisms that underlie these physical appearance traits gradually have been revealed. For example, feather-crested head is a prominent feature exhibited in several breeds of chicken, such as Silkies and Beijing-You chickens. A previous study has shown that the crest phenotype is associated with ectopic expression of the *HOXC8* gene, which is located on the E22C19W28 linkage group, is expressed in cranial skin, and was identified using linkage analysis, genome-wide association, and expression analysis from 26 different crested and noncrested breeds (Wang *et al.* 2012).

For the feathered feet trait, two loci were identified in a previous study (Somes 1992). Other feather traits were investigated by Noorai *et al* (2012), who found that the rumpless and ear-tufted traits loci were located on chicken chromosomes 2 and 15, respectively. The positional candidate genes, *Irx1* and *Irx2* for the rumpless trait, and *TBX1* and *GNB1L* for the ear-tufted trait, were identified through genome-wide association and haplotype analyses.

A major quantitative trait locus (QTL) for bisexual expression of comb mass and several QTL specific to female comb mass have been identified (Wright *et al.* 2008). Linkage analysis and identical-by-descent mapping proved that copy number variation of intron 1 of the *SOX5* gene causes the pea-comb phenotype in chickens (Wright *et al.* 2009). In a further study, a locus that affects comb mass has been identified using three separate intercrosses between wild and domestic chickens. The locus contains two tightly linked genes, *BMP2* and *HAO1*, which together produce the range of pleiotropic effects observed (Johnsson *et al.* 2012). For the duplex-comb trait, Dorshorst *et al.* (2015) found that a genomic 20-kb duplication contains regulatory elements that affect EOMES expression in the embryonic chicken comb and two duplex comb phenotypes (V-shaped and Buttercup).

The Beijing-You (BJY) chicken is a color-feathered, slow-growing Chinese indigenous breed with a feather-crested head, beard and feathered feet (Zheng 1988). In a previous study, an F2 resource population was constructed from a cross between BJY and a commercial rapidly growing broiler line (Cobb-Vantress; CB). This population was used to identify loci and candidate genes for meat quality traits (Sun *et al.* 2013a), shank length (Sun *et al.* 2013b) and, polydactyly (Sun *et al.* 2014) through genome-wide association studies (GWAS) or combined GWAS and linkage analysis (LA) methods.

In this study, we used the linkage analysis for six physical appearance traits (feather-crested head, beard, comb weight, wattles weight and length, and feathered feet) to provide insight into their genetic basis (supporting information, File S1, File S2, File S3).

## Materials and Methods

### Animals and phenotypes

This research complied with the Guidelines for Experimental Animals established by the Ministry of Science and Technology (Beijing, China). The Chinese Academy of Agricultural Science F2 chicken population was described in previous studies (Sun *et al.* 2013a,b; Sun *et al.* 2014; Liu *et al.* 2015). It was derived from a cross between BJY and CB chickens.

In this study, a total of 400 chickens were used from three generations, which included 367 F2 chickens from 20 full-sib families in five batches. Blood was collected into acid citrate dextrose anticoagulant tubes from the brachial vein on d 56. At 93 d, F2 chickens were weighed and killed by stunning and exsanguination. Before they were killed, the feather-crested head, beard, and feathered feet traits of each chicken were recorded. The comb and wattles of each chicken were weighed after they were killed. The length of the wattles between the upper and the lower point of the organ were measured with a micrometer.

### Genotyping and single-nucleotide polymorphism (SNP) quality

Genomic DNA was extracted from blood samples using the phenol-chloroform method and 50 ng/µL were used for genotyping with the Illumina 60K Chicken SNP Beadchip. This was performed by DNA LandMarks Inc., Saint-Jean-sur-Richelieu, PQ, Canada, and is detailed in a previous study (Sun *et al.* 2013a). A total of 39 samples were excluded due to sample call rate <90% and 13,985 SNPs were removed for failing to meet one or more of the following conditions: SNP call rate <90%, minor allele frequency <3%, Hardy-Weinberg equilibrium test *P* of < 10^−6^ and SNPs with no assigned chromosome or linkage group. After quality control measures, 42,585 SNPs distributed among 28 chromosomes, linkage group (LGE22), and the Z chromosome, were used for analysis. The average physical distance between two neighboring SNPs was approximately 20.4 kb (Sun *et al.* 2013a; 2014).

### Linkage analysis

The genetic linkage map was constructed using 19 full-sib families (six males and 12 females from F0, five males and 20 females from F1, and 148 males and 152 females from F2). A total of 42,585 SNPs were analyzed with the improved version of CRI-MAP (2.503a, run in a 64-bit Unix system), which has been described in previous studies (Groenen *et al.* 2009; Elferink *et al.* 2010; Sun *et al.* 2014). The total length of the sex-average map was 3040.8 cM and the recombination rate of the map was 2.9 cM/Mb (Sun *et al.* 2014).

To reduce the effect of linkage disequilibrium on the results, 6518 independent SNPs were acquired in all autosomal chromosomes using the indep-pairwise option, with a window size of 25 SNPs, a step of five SNPs, and r^2^ threshold of 0.2 (Sun *et al.* 2013a). The independent SNPs were used for a QTL analysis of the physical appearance traits. A least-squares regression model was used for single-QTL analysis in the web-based software GridQTL (Allen *et al.* 2012). This included the fixed effects of sex, hatch, and family, along with additive and dominance coefficients for the putative QTL. Significance thresholds were calculated with a permutation test (Churchill and Doerge 1994). A total of 10,000 permutations were computed to determine 5% and 1% chromosome-wide significance levels. The 5% and 1% genome-wide significance levels were calculated following the Bonferroni correction:

$${\text{P}}_{\text{genome-wide}}=\text{1}-{\left({\text{1 - P}}_{\text{chromosome-wide}}\right)}^{\text{Ga}/\text{Gc}}$$

where Ga is the length of the genetic map of each chromosome and Gc is the length of the genetic map of all autosomal chromosomes. Confidence intervals for QTL positions were estimated by bootstrapping, as presented by Visscher *et al.* (1996).

## Results

### Traits statistics

We recorded and measured six physical appearance traits, which included three qualitative traits (feather-crested head, beard, and feathered feet) and three quantitative traits (comb weight, wattles weight, and length), in the F2 chicken population. χ^2^ results for the qualitative traits and descriptive statistics for the quantitative traits are shown in Table 1 and Table 2, respectively. For the qualitative traits, the observed ratio between F2 chickens with traits and those without traits deviated significantly from the expected 3:1 ratio (*P* < 0.05) (Table 1). For the quantitative traits, the comb and wattles traits showed large phenotypic variation (the C.V. from 74 to 144%, Table 2).

**Table 1:** χ^2^ test for feather-crested head, beard, and feathered feet at 3:1 expected ratio of traits to nontraits in F2 chickens

Traits	N	No. of Traits	No. of Nontraits	χ2	*P*	DF
Feather-crested head	326	226	100	5.30	< 0.05	1
Beard	326	134	192	197.96	< 0.05	1
Feathered feet	326	188	138	51.30	< 0.05	1

DF, degree of freedom.

**Table 2:** Descriptive statistics for comb and wattles traits in F2 chickens

Traits	N	Mean	SD	C.V.	Min	Max
Comb weight, g	314	1.34	1.61	120.11	0.03	9.07
Wattles weight, g	296	0.77	1.11	144.20	0.02	6.28
Wattles length, mm	259	13.58	10.06	74.10	0.76	40.60

SD, standard deviation; C.V., coefficient of variation.

Johnson transformations (Minitab 15, Minitab Inc., Quality Plaza, PA) were implemented before analysis of the wattles length trait as the data deviated from normality (data not shown).

### QTL mapping results

The linkage analysis results for the physical appearance traits are shown in Table 3. The physical positions of the QTL were provided based on the SNPs markers physical positions in the genetic map (Sun *et al.* 2014). For six traits, 18 QTL were detected at 5% or 1% significance at the chromosome-wide level or significance at the 1% genome-wide level.

**Table 3:** Physical appearance trait QTL mapped in the chicken CAAS F2 resource population

N	Traits	GGA	Position, cM/Mb	Confidence Interval, cM/Mb	F-Ratio	AE	DE	PV
1	Feather-crested head	2	4/1.25	0.0-282.0/0.00-135.46	8.05*	−0.02 ± 0.04	0.24 ± 0.06	4.71
2	Feather-crested head	28	48/3.81	0.0-48.0/0.00-3.81	5.06*	0.03 ± 0.04	0.19 ± 0.06	2.76
3	Feather-crested head	LGE22*^a^*	5/0.51	0.0-10.0/0.00-0.51	68.84***	0.43 ± 0.04	0.30 ± 0.07	32.1
4	Comb weight	3	74/25.55	14.0-223.0/4.60-106.58	7.26*	−0.33 ± 0.12	−0.40 ± 0.16	4.29
5	Comb weight	7	52/22.13	12.0-101.0/3.52-36.52	6.36*	−0.25 ± 0.11	−0.44 ± 0.16	3.71
6	Comb weight	25	2/0.14	0.0-58.0/0.00-1.78	6.29**	0.41 ± 0.12	0.06 ± 0.19	3.66
7	Comb weight	27	49/4.39	7.0-49.0/1.87-4.39	4.93*	−0.0 ± 0.11	−0.53 ± 0.17	2.74
8	Beard	27	4/1.51	0.0 - 11.0/0.00-2.23	19.05***	0.22 ± 0.04	0.08 ± 0.06	11.2
9	Wattles weight	2	210/100.64	72.0-307.0/28.62-153.31	8.9*	0.19 ± 0.10	0.53 ± 0.14	5.63
10	Wattles weight	9	14/3.80	12.5-92.0/3.62-24.46	5.74*	−0.14 ± 0.10	0.46 ± 0.15	3.46
11	Wattles weight	25	55/1.67	0.0-56.0/0.00-1.69	5.71*	0.04 ± 0.09	0.49 ± 0.15	3.44
12	Wattles weight	27	2/1.37	0.0-48.0/0.00-4.07	8.44**	−0.37 ± 0.10	−0.11 ± 0.15	5.32
13	Wattles length	14	11/4.21	0.0-62.0/0.00-14.79	6.10*	−0.48 ± 0.85	−4.65 ± 1.38	4.18
14	Wattles length	27	1/1.23	0.0-21.0/0.00-2.92	9.53**	−3.96 ± 0.92	0.64 ± 1.36	6.79
15	Feathered feet	8	71/22.83	14.0-90.0/4.19-29.44	6.98*	−0.17 ± 0.05	−0.00 ± 0.07	3.99
16	Feathered feet	12	4/1.50	0.0-75.0/0.00-2.01	7.03*	−0.02 ± 0.04	0.24 ± 0.07	4.04
17	Feathered feet	13	55/1.56	48.0-57.0/13.40-16.20	53.94***	0.37 ± 0.04	−0.17 ± 0.06	26.9
18	Feathered feet	15	47/12.22	41.0-53.0/11.37-12.82	40.06***	0.33 ± 0.04	0.15 ± 0.06	21.4

* 5% chromosome-wide significance; **1% chromosome-wide significance; ***1% genome-wide significance. QTL, quantitative trail loci; CAAS, Chinese Academy of Agricultural Science; GGA, *Gallus gallus*; AE, additive effect ± SE; DE, dominance effect ± SE; PV, percent of phenotypic variance explained by the QTL. PV% = [(MSR - MSF)/MSR] × 100, where MSR-residual mean square in the reduced model, MSF-residual mean square in the full model.

a LGE22, LGE22C19W28_E50C23 linkage group.

### Crest and comb

Three QTL for feather-crested head were detected on chromosomes *Gallus gallus* (GGA)2, GGA28, and linkage group LGE22. One QTL located at 5 cM/0.51 Mb on LGE22 was significant at the 1% genome-wide level and explained 32.1% of the phenotypic variation. The other two QTL (located at 4 cM/1.25 Mb on GGA2 and 48 cM/3.81 on GGA28, respectively) were significant at the 5% chromosome-wide level and explained from 2.76 to 4.71% of the phenotypic variation.

For the comb weight trait, four QTL were detected on GGA3, GGA7, GGA25, and GGA27. These QTL explained between 2.74% and 4.29% of the phenotypic variation. One QTL explained 3.66% of the phenotypic variation and was located at 2 cM/0.14 Mb on GGA25, with 1% chromosome-wide significance level. Three QTL with 5% chromosome-wide significance level were located on GGA3 (74 cM), GGA7 (52cM), and GGA27 (49 cM).

### Beard and wattles

One QTL for the beard trait was detected at 4 cM/1.51 on GGA27, with a 1% genome-wide significance level. It explained 11.2% of the phenotypic variation. For the wattles traits, a total of six QTL were detected on five chromosomes and explained from 3.44 to 6.79% of the phenotypic variation. Two QTL, with a 1% chromosome-wise significance level, were detected on GGA27. The QTL for wattles weight was detected at 2 cM/1.37 Mb, whereas the QTL for wattles length was detected at 1 cM/1.23. They explained 5–6% of the phenotypic variation. The other four QTL, with 5% chromosome-wide significance level were located at 210 cM/100.64 Mb on GGA2, 14 cM/3.80 on GGA9, 11 cM/4.21 Mb on GGA14, and 55 cM/1.67 Mb on GGA25. These QTL explained approximately 3–5% of the phenotypic variation.

### Feathered feet

For the feathered feet trait, four QTL were detected on GGA8, GGA12, GGA13, and GGA15. Two QTL explained more than 20% of the phenotypic variation and are located at 55 cM/1.56 Mb on GGA13 and 47 cM/12.22 Mb on GGA15 with a 1% genome-wide significance level. The other two QTL explained approximately 4% of the phenotypic variation and are located at 71 cM/22.82 on GGA8 and 4 cM/11.50 Mb on GGA12 with 5% chromosome-wide significance level.

## Discussion

In this study, the observed ratios of traits to non-traits for the feather-crested head, beard and feathered feet traits deviated significantly from the expected 3:1 ratio. These traits showed segregation in the F2 chicken population. The three quantitative traits, including comb weight, wattles weight and length traits, showed large phenotypic variations. The segregation and large variations in the F2 chicken population were used in the linkage analysis to identify the QTL or genes.

### Crest and comb

In chickens there are two feather crests phenotypes: the helmet-shaped, forward-inclining feather crests and the full crests (Bartels 2003). Beijing-You chickens have full crests. Previous research has shown that homozygous crested chickens are distinguishable from heterozygous chickens by the lack of filoplumes in the crest. The full crest in the domestic chicken is encoded by an incompletely dominant autosomal gene (Bartels 2003). Through further linkage analysis and GWAS, Wang *et al.* (2012) found that the crest gene is located in the LGE22. LGE22 of chicken corresponds to an orthologous region on human chromosome 12 (HSA12) near the 50 Mb region. The region between 45 and 60 Mb on HSA12 shows patches of conserved synteny with other parts of the chicken genome, including GGA1, GGA2, GGA7, and ChrUn_random; the latter consists of contigs that could not be localized to any specific chromosome. Expression analysis of tissues from crested and noncrested chickens showed that the crest is caused by a cis-acting regulatory mutation that affects the ectopic expression of *HOXC8*, which was in chicken genome ChrUn_random and in HSA12. In this study, We used linkage analysis to identify three QTL for the feather-crested head trait, especially one QTL with 1% genome-wide significance, at 5 cM/0.51 Mb on LGE22 with a 10-cM confidence interval, approximately 0.51 Mb (from 0.00 to 0.51 Mb) in physical map. There were one microRNA and seven genes; *MIR1668*, *LARP4*, *COX14*, *ACCN2*, *HDAC7*, *TWIST3*, *SLC48A1*, and *NEUROD4*, in this region. The 0.51-Mb region is likely very closely linked to the *HOXC8*. The 0.51 Mb (0.00−0.51 Mb) region could be important region for crest traits and should undergo further studies. The QTL at 4 cM/1.25 on GGA2 and 48 cM/3.81 on GGA28 have minor chromosome-significant levels and without some confirming data, which are very likely to be false positives.

In the domestic chicken there are three major variants of comb type; Rose-comb, Pea-comb, and Duplex-comb. The QTL or genes responsible for the comb type and weight traits have being gradually revealed (Wright *et al.* 2008, 2009; Sato *et al.* 2010; Johnsson *et al.* 2012; Dorshorst *et al.* 2015). More information about comb weight has been provided through linkage analysis in this study. One QTL for comb weight was detected at 74 cM/25.55 Mb on GGA3, which partly overlaps with a region identified in a previous study (at 57.2 cM, from 46.5 to 75.5 cM; Wright *et al.* 2008). An expressed sequence tag *BU105297* identified by Boardman *et al.* (2002) was located at this QTL peak. This QTL could be an important region and requires further studies. Another three QTL; at 52 cM/22.13 Mb on GGA7, 2 cM/0.14 Mb on GGA25, and 49 cM/4.39 on GGA27, also were identified in this study. These may represent new QTL for comb weight and also require further analysis.

### Beard and wattles

Beards are feather modifications that appear in domestic chickens and are composed of elongated contour feathers that project downward under the chin (Bartels 2003). In this study, one QTL for the beard trait was detected in a 4-cM/1.51-Mb region (confidence interval, 0.0−11.0 cM/0.00−2.23) on GGA27. The location of this 11 cM QTL region was approximately 2.23 Mb (0.00−2.23 Mb) in the physical map, which partly overlaps with a region for wattles weight (0.00−4.07 Mb) and length traits (0.00−2.92 Mb) identified in this study. The 2.23-Mb region partly overlaps with a region for chicken body composition (Ankra-Badu *et al.* 2010). There were four microRNAs and 22 genes, especially two growth and development related genes, wingless-type MMTV integration site family, member 3 (*WNT3*) and growth hormone (*GH*) genes in this region. A previous study found that the size of the wattles was reduced by the presence of a beard (Bartels 2003). These data indicate that the beard and wattles traits could have the same genetic basis. The 2.23-Mb region on GGA27 could be an important candidate region for beard and wattles traits in chicken.

### Feathered feet

In many breeds of domestic chicken, such as the Silky and Beijing-You chicken, a mutative transformation of scales to feathers on the tarsus, feet and toes, the so-called feathered feet trait (ptilopody), has become a breed characteristic (Bartels 2003). In a previous study, two QTL for the feathered feet trait were identified (Somes 1992), but the physical positions of these two QTL were not found. In this study, two QTL regions, 55 cM/1.56 Mb (48.0−57.0 cM, 2.80 Mb region from 13.40 to 16.20 Mb) on GGA13 and 47 cM/2.22 (41.0−53.0 cM, 1.45 Mb region from 11.37 to 12.82 Mb) on GGA15, for the feathered feet trait were identified, at the 1% genome-wide significance level. A total of one microRNA and 33 genes were in the 2.80 Mb region on GGA13. Especially, the follistatin-like 4 (*FSTL4*) gene was located at the QTL peak. There were one microRNA *MIR1464* and five genes (*C15H12ORF49*, *TBX3*, *TBX5*, *RBM19*, and *SDSL*) in the 1.45-Mb region on GGA15. Therefore, the 2.80-Mb region (13.40−16.20 Mb) and the 1.45-Mb region (11.37−12.82 Mb) could be important candidate regions for the feathered feet trait and require further investigation. Two QTL, at 71 cM/22.82 Mb on GGA8 and 4 cM/11.50 Mb on GGA4, for the feathered feet trait, also were identified in this study. Because the two QTL have minor chromosome-significant levels, they are very likely to be false positives and need further study.

In conclusion, the candidate regions and genes for the six physical appearance traits were identified through linkage analysis in chickens. The candidate regions and genes include a 10-cM/0.51-Mb region (0.0−10.0 cM/0.00−0.51Mb) on LGE22 for crest trait, the QTL at 74 cM/25.55 Mb on GGA3 for comb weight, the 11 cM/2.23 Mb region with *WNT3* and *GH* on GGA27 for beard and wattles traits, and the 9 cM/2.80 Mb region (48.0−57.0 cM/13.40−16.20 Mb) on GGA13 and 12 cM/1.45Mb region (41.0−53.0 cM/11.37−12.82 Mb) on GGA15 for the feathered feet trait. These candidate regions and genes provide more genetic information on physical appearance traits in the chicken.

## 
